# Factors Influencing Wasting in Children Under 5 in Arid Regions of Kenya

**DOI:** 10.1111/mcn.70036

**Published:** 2025-04-23

**Authors:** Dickson A. Amugsi, Estelle Sidze, Faith Thuita, Valerie L. Flax, Calistus Wilunda, Linda Adair, Bonventure Mwangi, Esther Anono, Hazel Odhiambo, Stephen Ekiru, Gillian Chepkwony, Webale Albert, Ng'ang'a Monica, Joshua D. Miller, Bradley Sagara, Elizabeth Kimani‐Murage, Chessa Lutter

**Affiliations:** ^1^ African Population and Health Research Center, APHRC Campus Nairobi Kenya; ^2^ RTI International Research Triangle Park North Carolina USA; ^3^ Department of Public and Global Health University of Nairobi Nairobi Kenya; ^4^ Department of Nutrition University of North Carolina at Chapel Hill Chapel Hill North Carolina USA; ^5^ Mercy Corps Portland Oregon USA

**Keywords:** acute malnutrition, arid and semi‐arid, infants and young children, longitudinal, risk factors

## Abstract

Child wasting is a major public health problem in low‐ and middle‐income countries. Our study aimed to identify immediate, underlying and basic factors influencing wasting among children in Turkana and Samburu, two arid and semi‐arid regions in Kenya. Data are from a longitudinal study of children under 3 years of age at baseline, with follow‐up every 4 months for 2 years. Generalized estimating equations were used to assess risk factors of wasting in this population. Among immediate factors, children who recently experienced diarrhoea had 19% and 23% higher odds of wasting, and those who consumed animal‐source foods had 12% and 22% lower odds of wasting in Turkana and Samburu, respectively. Among underlying factors, children in Turkana whose caregivers used alcohol had 32% higher odds of wasting, whereas there was no effect of household food insecurity or factors related to water and sanitation on wasting in either county. Children in Turkana whose caregivers had 3–5 or 6 or more children had 39% and 70% higher odds, whereas those in female‐headed households had 34% and 81% higher odds of wasting in Turkana and Samburu, respectively. Male children also had increased odds of wasting; 21% and 41% in Turkana and Samburu, respectively. Children in Turkana's fisherfolk communities had 36% higher odds of wasting compared with those in urban or peri‐urban areas. Key risk factors for wasting included child sex, reported diarrhoea, caregiver's use of alcohol (in Turkana), caregiver's number of children, female‐headed households and fisherfolk livelihood (in Turkana) while consuming animal‐source foods was associated with lower risk. Interventions should target these intersecting factors to reduce wasting in these counties.

## Summary

- The prevalence of wasting among children aged less than 5 years was 22.8% at baseline in Turkana and 23.3% in Samburu counties, and changed little over our 2‐year study.
- Among immediate factors, children who had diarrhoea were more likely to be wasted, while those who consumed animal‐source foods were less likely to be wasted.
- Among underlying factors, children whose caregivers used alcohol were more likely to be wasted in Turkana. Household food insecurity and water, sanitation and hygiene were not associated with wasting.
- A variety of demographic factors were associated with higher likelihood of wasting, including male sex, number of children in the household, household being headed by a female, and fisherfolk livelihood (in Turkana).

## Graphical abstract

The analytical framework of factors influencing childhood wasting, illustrating the pathways involved in modelling these influences.

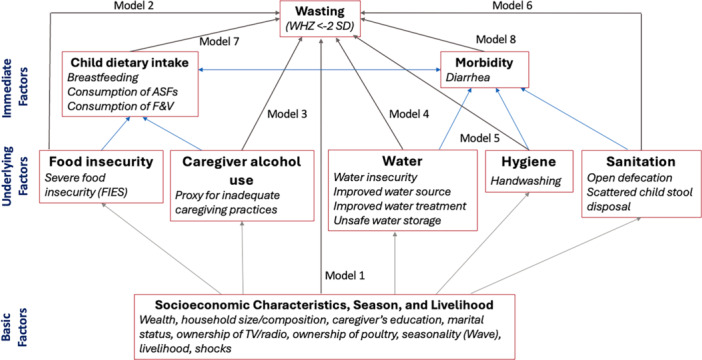

## 1 Introduction

Wasting is a critical global health issue characterized by a child having low weight for height. This condition disproportionately impacts children under 5, affecting an estimated 45.4 million children worldwide (WHO 2021) and contributing to approximately 45% of all preventable deaths in this age group (Ghosh‐Jerath et al. 2017; Bollinger and Trehan 2016). The prevalence of wasting is highest in low‐ and middle‐income countries, particularly in Sub‐Saharan Africa and South Asia (Mena et al. 2018). In Sub‐Saharan Africa, the number of undernourished people has risen from 5.5 million to 30 million in the last decade, resulting in the deaths of more than 3.5 million children under 5 annually (Owolade et al. 2022). In the Horn of Africa, over 7 million children under 5 are currently undernourished and in urgent need of nutrition support, with 1.9 million at risk of wasting‐related mortality (UNICEF 2023). Identifying risk factors of wasting has the potential to inform the development of more effective interventions to reduce its burden.

In Kenya, undernutrition – including stunting, wasting and underweight – among children is a persistent public health challenge (Mbogori & Muriuki 2022; KNBS and ICF 2023). Although there has been progress toward improved child nutritional status in recent decades (Mbogori & Muriuki 2022), the pace has been slow. For example, the prevalence of wasting decreased by only 3 percentage points in the past 30 years (Mbogori & Muriuki 2022). At the subnational level, the situation is particularly severe in Kenya's arid and semi‐arid regions, including Turkana and Samburu counties. The prevalence of child wasting in these areas is among the highest in the country, averaging 24% (MOH 2023) compared to the national average of 5% (KNBS and ICF 2023). Environmental, socioeconomic and cultural factors contribute to these observed disparities in the prevalence of wasting (Adepoju and Allen 2019; Hawkes et al. 2017).

In Turkana and Samburu, 60% and 57% of households rely on pastoralism as their primary livelihood, which limits access to diverse diets (Kumssa et al. 2009). Limited economic opportunities, inadequate security and lack of basic services exacerbate the vulnerability of these populations (Kumssa et al. 2009). Additionally, resource scarcity, including limited water and grazing land, often leads to violent conflicts among pastoralist communities, disrupting access to essential resources and worsening food security (McCrone 2023) with a consequential effect on child nutrition outcomes. Environmental degradation due to overgrazing, deforestation and climate change further diminishes the availability and quality of foods (Ogalo and Onyango 2016; Okello et al. 2009). Economic constraints also limit the ability to afford nutritious diets across all livelihoods in these counties (Save the Children 2017), despite caregivers generally being knowledgeable about infant and young child feeding recommendations (GAIN, USAID, Adesa 2016). In addition to widespread poverty, low literacy and historic underinvestment, Turkana and Samburu Counties also experience significant challenges with infrastructure, healthcare access and education, each of which negatively affects child nutrition and well‐being (Okello et al. 2009). Gender inequality compounds these issues, as women are often responsible for fetching water, collecting firewood and preparing meals, leaving limited time for childcare and feeding (Christian et al. 2023). There is also limited access to safe and functional water, sanitation and hygiene (WASH) services, increasing the risk of infectious diseases, such as diarrhoea, which impairs nutrient absorption, resulting in child undernutrition (Christian et al. 2023).

Despite the complex, multifaceted factors driving wasting in these counties, most studies have been cross‐sectional, limiting the ability to capture dynamic changes in risk of wasting across time. To address this gap, we conducted a 24‐month longitudinal study to identify risk factors for wasting among young children in Turkana and Samburu Counties. This design allows for a more comprehensive understanding of how social, economic and environmental factors vary and interact to influence child wasting risk. We aimed to identify key contextual factors influencing wasting in these regions. Such information will help inform contextually relevant policies and programmes to mitigate wasting in arid and semi‐arid areas.

## 2 Methods

### 2.1 Study Setting

This study was carried out as part of the USAID Nawiri programme and was intended to contribute to programme design and adaptation. The study was conducted from April 2021 to October 2023 in Turkana and Samburu Counties, located in Kenya's North Rift. These arid, resource‐scarce regions are mainly inhabited by nomadic pastoralist and agro‐pastoralist communities. The counties face numerous challenges, including severe droughts, limited access to essential services such as healthcare and education, and frequent intercommunal conflicts over scarce resources. Additionally, the counties have a high poverty rate and limited access to improved infrastructure (e.g., safely managed drinking water services and improved hygiene services), hindering economic growth and human development.

### 2.2 Study Sample

A detailed explanation of the design, sample size estimation and sampling strategy is described elsewhere (Wilunda et al. 2024). In brief, a multistage sampling strategy was used to ensure that findings were representative at both the county and sub‐county levels. In the first stage, the population was stratified according to survey zones, as defined by respective county governments. Within each survey zone, villages were randomly selected for inclusion. Locally hired field enumerators then conducted a household listing in the selected villages to create a sample frame of households with children under 3 years of age. In the final stage, households were randomly chosen from this sampling frame for participation in the study. This sampling approach was designed to achieve enough completed interviews to estimate key indicators with acceptable precision. All children aged 3 years or younger, along with their biological mothers or caregivers, were eligible to participate.

### 2.3 Data Collection

At enrolment (Wave 1), surveys were administered to collect information about household sociodemographic characteristics and individual health and nutrition behaviours and status. During subsequent survey waves, only health and nutrition information was collected.

We collected data on household characteristics, such as drinking water service, type of toilet facility, type of cooking fuel, asset ownership and experiences with various shocks in the last 4 months. Household food insecurity was assessed using the eight‐item Household Food Insecurity Experience Scale (FIES) (Wambogo et al. 2018). Household water insecurity was measured using the 12‐item Household Water Insecurity Experiences (HWISE) Scale (Young et al. 2019). Participants indicated the frequency with which they encountered difficulties accessing or using water for domestic purposes over the previous 4 months. Their responses were aggregated, yielding a score between 0 and 36. Based on these scores, households were classified into the following categories: no to marginal (0–2), low (3–11), moderate (12–23) and high‐water insecurity (24–36) (Frongillo et al. 2024).

At the individual level, eligible mothers or caregivers provided sociodemographic information, such as age, education, marital status, number of children, and sex of the household head. Caregivers also reported on child feeding practices, including breastfeeding and complementary feeding on the prior day, using questions recommended by the World Health Organization and UNICEF (WHO and UNICEF 2021). Health status was assessed by asking caregivers about common illnesses, such as diarrhoea and respiratory infections, experienced by the child in the 2 weeks before the survey.

Child weight was measured using electronic Seca scales, which were designed and produced under the guidance of UNICEF. Length (for children under 2 years) and height were measured using measuring boards produced by Shorr Productions. Two measurements for both weight and height were taken, and the average was used for analysis. Height and weight data were converted into *Z*‐scores based on the 2006 WHO growth standards (WHO 2006).

The target sample sizes were 1544 for Turkana and 669 for Samburu, but baseline data were collected from 1211 participants in Turkana and 586 in Samburu. For this analysis, we included 1201 children from Turkana and 582 from Samburu with plausible anthropometric data (weight‐for‐height *Z*‐scores between −5 SD and +5 SD) and complete data for relevant covariates.

### 2.4 Outcome Variable

The primary outcome of this analysis was wasting. Children were categorized as being wasted if their weight‐for‐height *Z*‐scores were below −2 standard deviations or not wasted if their weight‐for‐height *Z*‐scores were greater than or equal to −2 standard deviations.

### 2.5 Explanatory Variables

Independent variables were selected based on a conceptual framework for acute malnutrition in Africa's drylands, which categorizes contributing factors into three levels: immediate, underlying and basic (Figure S1, Young 2020). Immediate factors included child diarrhoea in the prior 2 weeks and consumption of breastmilk, animal‐source foods (meat, fish, eggs or dairy products), and fruits and vegetables (all types) in the prior day, each coded as dichotomous (yes/no).

Underlying factors included food insecurity, caregiver behaviours and WASH variables. Household food insecurity was assessed using the FIES, developed by Ballard et al. (2013). This scale includes eight questions designed to measure varying levels of food insecurity severity. Respondents answered each question with a simple yes or no, which provides insights into their access to sufficient, safe and nutritious food. The responses were then categorized as no‐to‐mild and moderate‐to‐severe food insecurity (Ballard et al. 2013). Self‐reported caregiver alcohol consumption (yes/no) was considered a proxy for inadequate caregiving practices. Household water insecurity was categorized as no‐to‐marginal, low, moderate or high. Other factors included water source, categorized as improved and unimproved. Improved water sources included household connections, public standpipes, boreholes, protected dug wells, protected springs and systems for rainwater collection. In contrast, unimproved water sources encompass unprotected wells and springs, surface water such as rivers and lakes, water supplied by vendors, and water delivered by tanker trucks. Sanitation facilities were similarly classified into improved and unimproved categories. Improved sanitation facilities consisted of flush or pour‐flush toilets connected to sewer systems, septic tanks, ventilated improved pit latrines, and pit latrines with slabs. Unimproved sanitation facilities include shared toilets, flush toilets that drain improperly, pit latrines without slabs, bucket latrines and hanging latrines. Other variables included treatment of drinking water (yes/no), self‐reported caregiver handwashing after toilet use (yes/no), household open defecation (yes/no) and disposal of the youngest child's stool (safe/unsafe).

Basic factors included household size (< 4, 4–6, 7+ members), household ownership of poultry (yes/no) or a television or radio (yes/no), and wealth index. The household wealth index was developed using principal component analysis based on the baseline (Wave 1) data. This involved assessing various factors, such as the ownership of specific assets (e.g., refrigerator, radio, TV, motorcycle, car/truck, etc.), the materials used for walls, floors and roofs, and the types of lighting sources present in each household. By integrating these elements, we aimed to establish a comprehensive measure of wealth that reflects the living conditions of the households. Once the index was computed, it was utilized to categorize households into distinct wealth groupings, specifically into tertiles (low, medium, high). This categorization helps to provide a clearer understanding of wealth distribution within the studied population. The sex of the household head (male or female) and caregiver marital status (unmarried, married and only wife, married and a co‐wife) were included, along with the caregiver's parity (0–2, 3–5, 6+ children), age (< 25, 25–34, 35+ years) and educational level (formal or no formal education). Respondents also reported whether they were the biological mother of the child (yes/no). Additional variables included livelihood (urban/peri‐urban, pastoral, agropastoral, fisherfolk), survey zone (East, North, South, West) and survey wave, which served as a proxy for seasonality. Shocks were categorized as climatic, economic, biological or conflict, and analyzed as the total number across categories (0–1, 2, 3, 4). Child‐specific variables, such as age and sex, were considered.

### 2.6 Data Analysis

**Figure 1:**
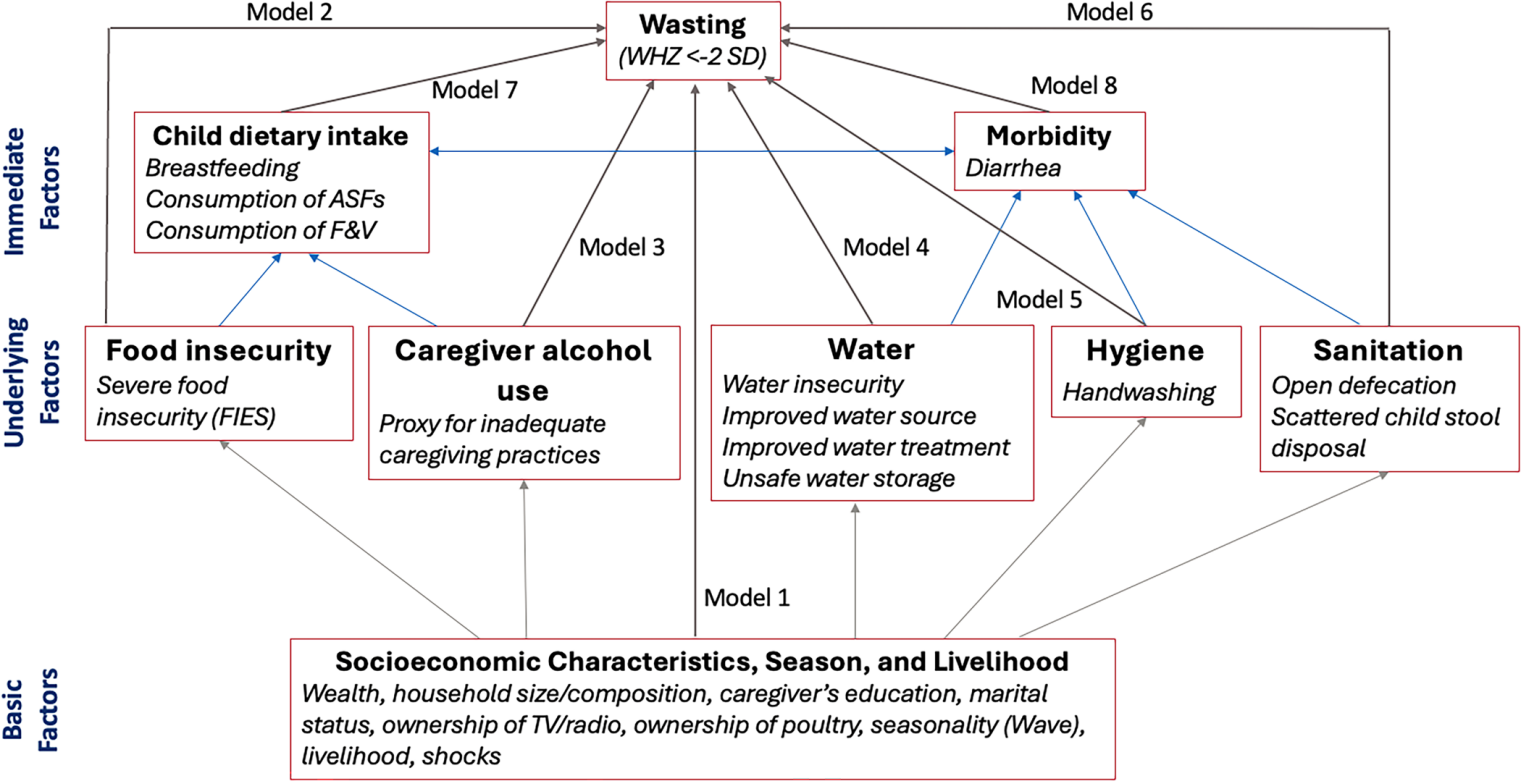
Analytical framework of drivers of wasting illustrating modelling pathways.

To guide our analysis, we developed an analytic framework **(**Figure 1 **)** that aligns with the conceptual framework for acute malnutrition in Africa's drylands (Young 2020). For each level, we selected variables that most accurately captured key factors outlined in the theoretical model. We then added other variables based on their theoretical importance given the current literature, parsimonious representation of key concepts (e.g., use of a validated scale for food insecurity or indicators that capture multiple exposures), and their associations with the outcome variable.

We employed generalized estimating equations to assess the effects of immediate, underlying and basic factors on wasting, using pooled data from all six survey waves. The generalized estimating equations assumed a binomial distribution, a logit link function, an exchangeable correction matrix, and a robust variance estimator. After initial bivariate analyses to identify variables to include in multivariable analyses using a cutoff of *p* < 0.20, we built eight models to identify factors associated with wasting at *p* < 0.05 (Figure 1). In model 1, we assessed the associations between basic risk factors and wasting. In model 2, we examined the association between food insecurity and wasting. Model 3 examined the association of caregiver alcohol consumption with wasting. Models 4–6 examined the association between the WASH variables with wasting. Models 7 and 8 examined the associations of dietary intake variables and diarrhoea, respectively, with wasting. Models 2–8 adjusted for the same basic factors, including survey zone, livelihood zone, survey wave, and child's age and sex. Furthermore, we built a distinct breastfeeding model using data from children under 3 years old to assess the impact of breastfeeding on this specific age group. Data were analyzed separately for each county using Stata 18 (StataCorp, College Station, Texas, USA). The factors influencing sample selection (livelihood and survey zones) were accounted for in the models, ensuring that the standard errors were properly estimated.

### 2.7 Ethics Statement

The African Population and Health Research Centre (APHRC) obtained ethical and research approvals and research permits from Amref Health Africa's Ethical and Scientific Review Committee (Amref ESRC P905/2020) and the National Commission for Science, Technology, and Innovation of Kenya and signed a reliance agreement with RTI International's Institutional Review Board for the research.

## 3 Results

### 3.1 Characteristics of the Samples

At Wave 1, the prevalence of wasting was 21.8% in Turkana and 23.3% in Samburu (Table 1). In Turkana, 54.8% of the children were male, while in Samburu, 47.8% were male. Across counties, a plurality of children were 11 months old or younger (45.2% in Samburu, 39.7% in Turkana). Most caregivers had no formal education, were 25–34 years old, lived in male‐headed households, and resided in pastoral zones.

**Table 1:** Child, caregiver, household and community characteristics at Wave 1 by county.

Characteristic	Turkana (*N* = 1211) (%)	Samburu (*N* = 586) (%)
*Characteristics of children*		
Prevalence of wasting	21.8	23.3
Sex		
Male	54.8	47.8
Female	45.2	52.2
Age (Months)		
0–11	39.7	45.2
12–23	30.8	33.3
35+	32.6	14.7
*Characteristics of caregivers/mothers*		
Age (years)		
< 25	19.8	38.5
25–34	47.6	46.8
35 and above	32.6	14.7
Marital status		
Married only wife	50.1	54.2
Unmarried	14.8	14.3
Married co‐wife	35.1	31.4
Gravidity		
< 3	23.6	36.4
3–5	51.2	33.2
5 and above	25.1	30.3
Parity		
Less than 2	25.9	33.7
3–5	49.6	34.0
6 or more	24.4	32.3
Highest level of education		
Formal education	13.6	19.6
No formal education	86.4	80.4
*Characteristics of households*		
Age of the household head (years)		
< 25	10.8	11.3
25–34	34.5	36.3
35 and above	54.6	52.4
Sex of household head		
Male	61.9	83.7
Female	38.1	16.3
Household wealth tertile		
Lowest	45.6	55.7
Middle	33.8	30.5
Highest	20.6	13.8
Household size		
< 4	9.6	11.7
4–6	49.5	53.5
7+	40.9	34.8
*Community factors*		
Livelihood		
Urban/peri‐urban	6.5	11.4
Pastoral	63.9	83.4
Agropastoral	14.8	5.2
Fisherfolk	14.8	n.a.
Survey zone		
Central	21.0	7.1
North	25.6	77.1
South	24.1	n.a.
East	n.a.	15.8
West	29.2	n.a.

### 3.2 Trends in Wasting by County and Livelihood

**Figure 2:**
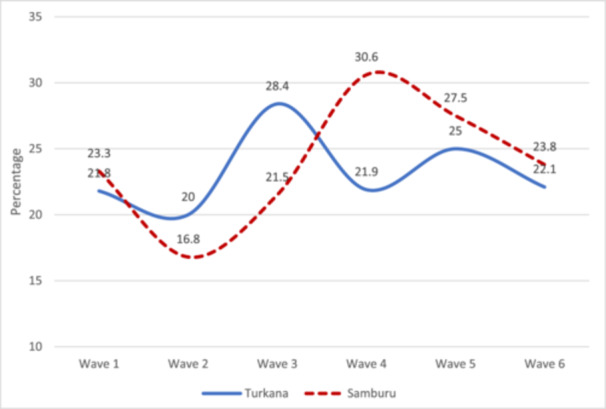
Trends in the prevalence of acute malnutrition (WHZ < −2 SD) among children by county, Turkana and Samburu. Wave 1, May–June 2021; Wave 2, November 2021; Wave 3, May–June 2022; Wave 4, October–November 2022; and Wave 5, March 2023; Wave 6: August–September 2023.

**Figure 3:**
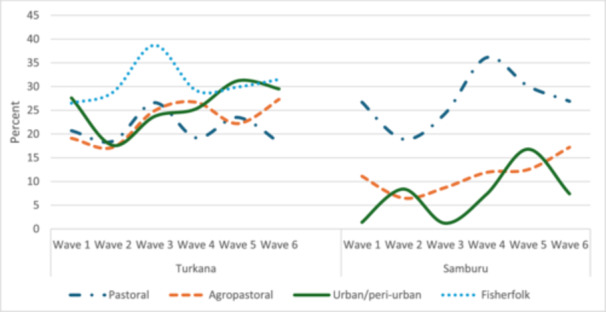
Trends in the prevalence of acute malnutrition (WHZ < −2 SD) among children by livelihood and county. Turkana: Wave 1, May–June 2021; Wave 2, November 2021; Wave 3, May–June 2022; Wave 4, October–November 2022; and Wave 5, March 2023; Wave 6: August–September 2023. Samburu: Wave 1: June/July 2021; Wave 2: November/December 2021; Wave 3: April/May 2022; Wave 4: October 2022; Wave 5: March/April 2023; Wave 6: August 2023.

Wasting prevalence among children under 5 was persistently high across survey waves, with no improvement from Wave 1 to Wave 6 (Figure 2). When examined by livelihood zone, wasting decreased from Wave 1 to Wave 6 among pastoralists (20.7%–18.2%), while there was an increase among agro‐pastoralists (19.1%–27.3%), fisherfolk (26.5%–31.5%) and urban/peri‐urban dwellers (27.6%–29.5%) in Turkana (Figure 3). In Samburu, the prevalence of wasting remained persistently high, showing no improvement in trends between Waves 1 and 6 (Figure 3). Although the prevalence in the agropastoral and urban/peri‐urban livelihoods was lower than among pastoralists, there was a general increase in the trends of wasting between Waves 1 and 6.

### 3.3 Basic Risk Factors of Wasting

In multivariable models of basic factors, female children, compared with male children, had 21% (aOR = 0.79, 95% CI = 0.65, 0.97) lower odds of wasting in Turkana and 41% (aOR = 0.59, 95% CI = 0.42, 0.82) lower odds in Samburu (Table 2). Child age was not associated with wasting. In Samburu, but not Turkana, children of caregivers with formal education had 44% (aOR = 0.56; 95% CI = 0.35, 0.88) lower odds of wasting than those whose caregivers did not have any formal education. In Turkana only, compared with children of caregivers younger than 20 years, children of caregivers aged 20–34 or 35 years or older had 38% (aOR = 0.62; 95% CI = 0.42, 0.92) and 42% (aOR = 0.58; 95% CI = 0.38, 0.90) lower odds of wasting, respectively. Children of caregivers with 3–5 or 6 or more children had 39% (aOR = 1.39; 95% CI = 1.12, 1.73) and 70% (aOR = 1.70; 95% CI = 1.30, 2.23) higher odds of wasting, respectively, compared with those with 2 or fewer children. Compared with children in male‐headed households, children in female‐headed households had 34% (aOR = 1.34; 95% CI = 1.07, 1.66) and 81% (aOR = 1.81; 95% CI = 1.07, 3.07) higher odds of wasting in Turkana and Samburu, respectively. Children in the fisherfolk livelihood in Turkana had 36% (aOR = 1.36; 95% CI = 1.01, 1.82) higher odds of wasting compared with those in urban or peri‐urban areas. The odds of wasting were 15% (aOR = 0.85; 95% CI = 0.72, 1.00) and 29% (aOR = 0.71; 95% CI = 0.53, 0.94) lower at Wave 2 than at Wave 1 in Turkana and Samburu, respectively.

**Table 2:** Associations between basic factors and wasting among children under 5 years of age in Turkana and Samburu.

	Turkana (*N* = 1201)	Samburu (*N* = 582)
Variable	aOR [95% CI]	aOR [95% CI]
*Characteristics of children*		
Female child (ref: male)	0.79 [0.65, 0.97]*	0.59 [0.42, 0.82]**
Child's age (ref: 0–1 year)		
1 year	1.11 [0.91, 1.36]	0.84 [0.61, 1.16]
2 years	1.01 [0.78, 1.30]	0.85 [0.57, 1.28]
3 years	1.06 [0.77, 1.48]	1.43 [0.84, 2.41]
4 years	1.38 [0.92, 2.07]	1.83 [0.96, 3.49]
*Characteristics of caregivers/mothers*		
Caregiver received formal education (ref: did not)	0.95 [0.76, 1.19]	0.56 [0.35, 0.88]*
Caregiver age (ref: < 20 years)		
20–34 years	0.62 [0.42, 0.92]*	0.77 [0.55, 1.08]
35+ years	0.58 [0.38, 0.90]*	0.72 [0.47, 1.10]
Caregiver marital status (ref: married, only wife)		
Unmarried	0.89 [0.73, 1.08]	1.11 [0.72, 1.72]
Married, co‐wife	0.86 [0.70, 1.07]	1.07 [0.73, 1.57]
Caregiver biological mother (ref: not)	1.02 [0.77, 1.35]	1.27 [0.69, 2.34]
Caregiver number of children (ref: 0–2 children)		
3–5	1.39 [1.12, 1.73]**	0.94 [0.65, 1.36]
6 or more	1.70 [1.30, 2.23]***	1.15 [0.75, 1.76]
*Characteristics households*		
Female household head (ref: male)	1.34 [1.07, 1.66]**	1.81 [1.07, 3.07]*
Household owns poultry (ref: does not)	1.10 [0.91, 1.33]	1.00 [0.68, 1.48]
Household owns TV or radio (ref: does not)	0.92 [0.71, 1.19]	0.89 [0.65, 1.22]
Wealth tertile (ref: highest)		
Lowest	1.19 [0.94, 1.51]	0.98 [0.61, 1.58]
Middle	1.03 [0.84, 1.27]	1.00 [0.68, 1.48]
Shocks (ref: 0–1 shocks)		
2 shocks	0.98 [0.79, 1.23]	0.80 [0.59, 1.07]
3 shocks	0.91 [0.73, 1.14]	0.75 [0.55, 1.01]
4 shocks	0.99 [0.78, 1.25]	0.74 [0.53, 1.05]
*Community factors*		
Survey zone (ref: central)		
East	n.a.	0.86 [0.52, 1.40]
North	1.12 [0.80, 1.58]	1.30 [0.81, 2.09]
South	1.15 [0.88, 1.50]	n.a.
West	0.88 [0.67, 1.18]	n.a.
Livelihood zone (ref: urban/peri‐urban)		
Pastoral	0.88 [0.76,1.03]	1.30 [0.93,1.82]
Agropastoral	0.83 [0.67,1.03]	1.11 [0.80,1.54]
Fisherfolk	1.36 [1.01,1.82]*	n.a.
Survey wave (ref: Wave 1)		
Wave 2	0.85 [0.72, 1.00]*	0.71 [0.53, 0.94]*
Wave 3	1.16 [0.96, 1.39]	0.82 [0.59, 1.12]
Wave 4	0.89 [0.72, 1.10]	1.11 [0.79, 1.57]
Wave 5	1.00 [0.80, 1.27]	0.96 [0.65, 1.42]
Wave 6	0.78 [0.59, 1.02]	0.86 [0.55, 1.33]

Abbreviations: 95% CI, 95% confidence interval; aOR, adjusted odds ratio; n.a., not applicable.

* *p* < 0.05;

** *p* < 0.01;

*** *p* < 0.001.

### 3.4 Immediate and Underlying Risk Factors of Wasting

Children who experienced diarrhoea in the 2 weeks before the surveys, compared with those who did not, had 19% (aOR = 1.19, 95% CI = 1.06, 1.34) and 23% (aOR = 1.23, 95% CI = 1.02, 1.47) higher odds of wasting in Turkana and Samburu, respectively (Table 3). Children who consumed animal‐source foods in the 24 h before the surveys, compared with those who did not, had 12% (aOR = 0.88, 95% CI = 0.78, 0.99) and 22% (aOR = 0.78, 95% CI = 0.65, 0.93) lower odds of wasting in Turkana and Samburu, respectively. In Samburu, the odds of wasting were 38% (aOR = 1.38, 95% CI = 1.05, 1.82) higher in children who received breastmilk compared with those who did not; this association was not significant when restricted to children younger than 3 years (*results not shown*). In Turkana, children of caregivers who used alcohol had 32% (aOR = 1.32, 95% CI = 1.10, 1.60) higher odds of wasting compared with those whose caregivers did not. Household food insecurity and the WASH variables (household water insecurity, caregiver hand washing and open defecation) were not associated with wasting in either county.

**Table 3:** Associations of immediate and underlying factors with wasting among children under 5 years of age in Turkana and Samburu.

	Turkana (*N* = 1201)	Samburu (*N* = 582)
Variables	aOR [95% CI]	aOR [95% CI]
Immediate factors		
Child diarrhoea (ref: no diarrhoea)	1.19 [1.06, 1.34]**	1.23 [1.02, 1.47]*
Consumed animal‐source foods (ref: did not)	0.88 [0.78, 0.99]*	0.78 [0.65, 0.93]**
Consumed fruits and vegetables (ref: did not)	0.88 [0.76, 1.01]	1.14 [0.93, 1.40]
Consumed breastmilk (ref: did not)	1.11 [0.96, 1.29]	1.38 [1.05, 1.82]*
Underlying factors		
Moderate‐to‐severe food insecurity (ref: no‐to‐mild food insecurity)	1.00 [0.83, 1.21]	1.01 [0.75, 1.34]
Caregiver consumes alcohol (ref: does not)	1.32 [1.10, 1.60]**	1.33 [0.81, 2.20]
Household water insecurity (ref: no‐to‐marginal)		
Low	1.05 [0.89, 1.23]	0.97 [0.74, 1.26]
Moderate	1.03 [0.90, 1.19]	0.89 [0.69, 1.16]
High	1.04 [0.88, 1.23]	1.12 [0.80, 1.57]
Caregiver wash hands after using toilet (ref: does not wash hands)	1.08 [0.94, 1.24]	1.11 [0.92, 1.33]
Open defecation (ref: no open defecation)	1.13 [0.96, 1.33]	1.16 [0.89, 1.53]

Abbreviations: 95% CI, 95% confidence interval; aOR, adjusted odds ratio.

* *p* < 0.05;

** *p* < 0.01.

## 4 Discussion

This longitudinal study examined trends and factors influencing wasting among children under 5 in two arid and semi‐arid counties in Kenya. Our descriptive analysis revealed that wasting remained high in Turkana and Samburu, showing no improvement over the 24‐month study period. These results, and prior work in this region (MOH 2023), suggest that wasting remains a priority issue in this area despite concerted efforts by government and development partners to address it. Additionally, we identified factors influencing both increased and reduced risk of wasting, findings that have practical implications for nutrition and multi‐sectoral interventions in these counties.

Among the examined basic factors, we found that children residing in households headed by women were at a higher risk of experiencing wasting compared with those living in households headed by men. While women often prioritize food security and dietary diversity, positively impacting children's nutrition (Addai et al. 2022; Smith et al. 2003), financial constraints and limited access to resources in this setting may hinder their ability to provide adequate nutrition, as previously found in Malawi (Chirwa and Ngalawa 2008). Children of caregivers with more than three children also had higher odds of wasting. Larger families may face challenges as household members must share limited resources, potentially resulting in smaller meals and inadequate nutrition (Shafiq et al. 2021). These findings underscore the need for targeted interventions to support female‐headed households and larger families, which could significantly improve children's nutritional status in these vulnerable groups.

Caregiver education was another important basic factor associated with wasting. In Samburu, children of caregivers with formal education had a lower risk of wasting compared with those whose caregivers lacked formal education, which aligns with prior literature (Iftikhar et al. 2017; Abdirahman et al. 2019; Ejike 2016). Formal education provides caregivers with knowledge and skills for informed decisions on child‐feeding practices, hygiene and healthcare utilization (Fadare et al. 2019). The influence of caregiver education on child nutrition, however, varies based on the local context and availability of other sources of nutrition‐related knowledge. Indeed, prior research has found that maternal education and nutrition knowledge are distinct predictors of child nutritional status (Webb and Block 2004), suggesting that targeted nutrition‐education interventions in areas with limited formal education, such as Turkana and Samburu, may help to reduce the burden of wasting (Fadare et al. 2019).

Livelihood was another salient predictor of child wasting. In Turkana, children in the fisherfolk livelihood had higher odds of wasting compared with those living in urban or peri‐urban areas. Although fish are a nutrient‐dense food often associated with improved child nutrition, fishing households may not have equitable access to the benefits of their catch, potentially due to a lack of investment and technical support (Fiorella et al. 2014). Further, the reliance on fishing as a livelihood strategy may divert time and resources away from other food‐production activities that support child nutritional well‐being (Nyawade et al. 2021). To overcome these challenges and fully harness the benefits of fishing as a livelihood strategy, it is essential to implement carefully designed policies and programmes that address the unique challenges faced by fishing communities in Turkana. This could involve targeted investments in infrastructure, such as improving storage facilities and access to clean water, as well as enhancing market access for fisherfolk. Capacity‐building programmes aimed at equipping fishing communities with the necessary knowledge and skills for sustainable fishing practices and diversified livelihood opportunities could also be beneficial. Furthermore, addressing the underlying social, economic and environmental factors that affect food security and nutritional outcomes in the region is crucial. This may entail initiatives to strengthen social safety nets and efforts that support food production to improve the nutritional outcomes of children in the fisherfolk zone.

Results disaggregated by child sex revealed that female children were less likely to experience wasting compared with their male counterparts, as observed in other studies. For instance, a recent systematic review and meta‐analysis conducted by Thurstans et al. (2020) found that in most contexts, boys are more likely to experience wasting, although there are notable regional variations, suggesting that social, genetic or environmental factors may influence these disparities in nutritional status. Similarly, a pooled analysis of 33 longitudinal cohorts from 15 low‐ and middle‐income countries (LMICs) identified the male sex as a significant predictor of both wasting and stunting (Mertens et al. 2023). The drivers of this observed inequality remain unclear, and it is not yet known whether they stem from biological factors – such as sex‐specific differences in immune function, metabolism or growth patterns – or social factors, including disparities in care practices, feeding behaviours or access to resources. As such, a comprehensive approach that addresses both physiological and socioeconomic factors contributing to wasting is necessitated.

Among examined immediate factors, diarrhoea was found to be associated with higher odds of wasting. This is probably because diarrhoeal diseases lead to significant losses of fluids and electrolytes, essential for maintaining the body's hydration and overall functioning. These conditions can also reduce appetite, making it difficult for children to consume enough food to meet their nutritional needs. Additionally, during episodes of diarrhoea, the body's ability to absorb nutrients from food is impaired, further increasing the risk of nutrient deficiencies, and leading to wasting. The stress of illness may also raise the body's demand for certain nutrients, heightening the risk of wasting (Brown 2003; Hodges et al. 2015; Weisz et al. 2011). On the other direction, malnutrition impairs immune function, increasing susceptibility to diarrhoea (Brown 2003). The well‐established bidirectional relationship between undernutrition and diarrhoea indicates that interventions must address both aspects simultaneously (Brown 2003).

Indeed, addressing the impact of diarrhoea on wasting necessitates a comprehensive and multifaceted approach. Enhancing WASH practices within communities is essential. This involves improving access to safe and reliable drinking water, promoting safe food handling techniques, and ensuring the availability of proper sanitation facilities to minimize the risk of diarrhoea (Nounkeu et al. 2021; Mwaniki and Makokha 2013). Additionally, it is critical to prioritize the timely treatment and management of diarrhoea. This includes the prompt use of oral rehydration solutions to prevent dehydration and providing zinc supplementation to help reduce the duration of diarrhoea episodes. Healthcare providers must be well‐equipped to monitor and manage severe cases effectively (Mwaniki and Makokha 2013). Moreover, interventions aimed at improving dietary intake are vital. This may involve educating caregivers about nutritional practices, facilitating access to nutrient‐rich foods, and implementing feeding programmes that support the specific needs of undernourished children (Nounkeu et al. 2021; Mwaniki and Makokha 2013). Enhancing the nutritional status of affected individuals is crucial in mitigating the adverse effects of diarrhoea on health and nutrition. Therefore, a coordinated effort integrating these elements is necessary to achieve lasting improvements in health outcomes and nutritional well‐being of children (Nounkeu et al. 2021; Mwaniki and Makokha 2013).

Certain aspects of a child's diet were also significantly associated with wasting. In both counties, animal‐source food consumption was associated with lower odds of wasting among children under 5. Numerous studies have demonstrated that animal‐source foods provide high‐quality proteins, vitamins and minerals essential for child growth and development (Chege et al. 2015; Dror and Allen 2011; White et al. 2021; Dror and Allen 2011). As such, interventions that promote animal‐source food consumption can potentially prevent wasting and promote children's overall health (White et al. 2021; Balehegn et al. 2019).

With respect to underlying factors, we found that caregiver alcohol consumption was associated with higher odds of child wasting. Alcohol use can restrict caregivers' abilities to provide adequate care, resulting in impaired judgement, reduced responsiveness and decreased ability to create a supportive environment that young children need for healthy growth and development (Phillips and Adams 2001). Children of caregivers who use alcohol may also be at a higher risk of experiencing neglect, abuse or instability at home, which can further compromise their well‐being (Burchinal 1999). Efforts to reduce caregiver alcohol use through education, counselling and support services are necessary to help reduce the burden of wasting in Turkana.

Our analysis unexpectedly revealed no significant association between WASH practices and rates of wasting in either Turkana or Samburu counties, despite documented evidence from our analysis that suggests poor WASH practices among this population. For instance, the reported rates of adequate drinking water treatment are only 5% to 12% in Turkana and 2% to 10% in Samburu. Additionally, drinking water is frequently stored in open containers, with prevalence rates ranging from 92% to 97% in Turkana and 81% to 99% in Samburu. In Turkana, handwashing with soap occurs in 52% to 65% of instances, while open defecation poses a serious concern, with rates between 81% and 92% in Turkana and between 76% and 86% in Samburu (USAID Nawiri 2022). The lack of association may be linked to households' challenging environmental conditions in these settings. Such circumstances can undermine even the most well‐intentioned short‐term efforts, complicating the multifaceted challenge of addressing the various factors contributing to child wasting. Consequently, without substantial improvements to the broader environmental context, WASH initiatives may not yield significant child health and development benefits. It is important to note that previous research has also shown no significant relationship between WASH factors and child nutrition outcomes. Studies from the WASH Benefits and SHINE trials have demonstrated that various household‐level WASH initiatives, such as improved pit latrines, standalone handwashing stations, and point‐of‐use water chlorination, do not significantly enhance child growth outcomes (Pickering et al. 2019).

### 4.1 Strengths and Limitations

A major strength of this study was its longitudinal design, which allowed us to examine which risk factors of malnutrition are most important during critical periods of child development. Additionally, we used generalized estimating equations, which allowed us to account for intra‐individual correlation, assessed numerous factors across different levels of our framework, and had large sample sizes with little attrition in both counties.

However, the study has limitations. Although the attrition rate in the study was low, it could compromise the robustness of some estimates. Furthermore, considering the nature of the analysis, we could not establish causality between the study variables. Thus, our conclusions are carefully restricted to statements about the associations between the explanatory and outcome variables. This study was designed to capture seasons by collecting data during different expected seasons. However, Waves 2–6 were considered a drought period with successive failed rains; therefore, the seasons were not as expected, thus limiting our ability to connect different factors that could contribute to wasting with season. Further, the limited variability in WASH practices in these counties may have precluded detection of their effect on wasting, despite WASH being critical factors of child outcomes. Despite these limitations, understanding the factors influencing wasting in Turkana and Samburu regions over time is crucial for effective intervention and programme design.

## 5 Conclusions

Our study highlights persistently high levels of wasting in Turkana and Samburu, Kenya, with several factors associated with greater risk, including female‐headed households, larger family size, lack of caregiver education, livelihood zone, caregivers' alcohol use, and recent experience of diarrhoea. Conversely, child consumption of animal‐source foods was associated with lower odds of wasting. Gender‐specific interventions and programmes could be implemented to address wasting in these vulnerable households. Targeted interventions to reduce child diarrhoea and caregiver alcohol use and to increase consumption of animal‐source foods could also help reduce wasting. Our findings underscore the need for multifaceted interventions that concurrently address multiple underlying, basic and immediate factors to effectively reduce wasting in these regions.

## Author Contributions

D.A.A., E.S., F.T., V.L.F., C.W., B.M., E.A., H.O., S.E., G.C., W.A., N.M., E.K.‐M., C.L. and B.S. performed the research. D.A.A., E.S., V.L.F., C.L., F.T. and B.S. designed the research study. C.W., W.A., B.M., E.A., H.O., S.E., E.K.‐M., G.C. and N.M. contributed essential reagents or tools. L.A., J.D.M. and B.M. analyzed the data. D.A.A. wrote the first draft of the paper. D.A.A., E.S., F.T., V.L.F., C.W., E.K.‐M., C.L., L.A. and J.D.M. critically reviewed the manuscript. All authors have read and approved the final manuscript.

## Conflicts of Interest

The authors declare no conflicts of interest.

## Supporting information


**Supplemental Figure 1.** A conceptual framework for acute malnutrition in Africa's drylands (Young, 2020).

## Data Availability

Data from this study are available to researchers through the APHRC's Microdata Portal (https://aphrc.org/microdata-portal/).
