# Supplementary material for: Factors Influencing Wasting in Children Under 5 in Arid Regions of Kenya
Source: Matern Child Nutr. 2025 Apr 23;22(1):e70036. doi: 10.1111/mcn.70036 (PMC12647969; doi:10.1111/mcn.70036)

**Supplemental Figure 1.** A conceptual framework for acute malnutrition in Africa’s drylands (Young, 2020)


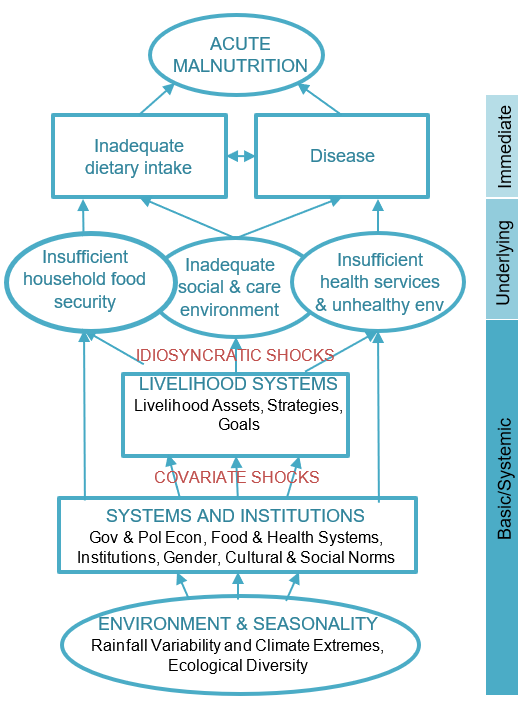

Supplement: Supplementary file 1 — Supplemental Figure 1. A conceptual framework for acute malnutrition in Africa's drylands (Young, 2020). [file MCN-22-e70036-s001.docx]
